# High-frequency actionable pathogenic exome variants in an average-risk cohort

**DOI:** 10.1101/mcs.a003178

**Published:** 2018-12

**Authors:** Shannon Rego, Orit Dagan-Rosenfeld, Wenyu Zhou, M. Reza Sailani, Patricia Limcaoco, Elizabeth Colbert, Monika Avina, Jessica Wheeler, Colleen Craig, Denis Salins, Hannes L. Röst, Jessilyn Dunn, Tracey McLaughlin, Lars M. Steinmetz, Jonathan A. Bernstein, Michael P. Snyder

**Affiliations:** 1 Department of Genetics, Stanford University School of Medicine, Stanford, California 94305, USA;; 2 Department of Medicine, Stanford University School of Medicine, Stanford, California 94305, USA;; 3 Mobilize Center, Stanford University, Stanford, California 94305, USA;; 4 Stanford Genome Technology Center, Stanford University, Palo Alto, California 94304, USA;; 5 European Molecular Biology Laboratory (EMBL), Genome Biology Unit, 69117 Heidelberg, Germany;; 6 Department of Pediatrics, Stanford University School of Medicine, Stanford, California 94305, USA

**Keywords:** diabetes mellitus, dilated cardiomyopathy, maturity-onset diabetes of the young

## Abstract

Exome sequencing is increasingly utilized in both clinical and nonclinical settings, but little is known about its utility in healthy individuals. Most previous studies on this topic have examined a small subset of genes known to be implicated in human disease and/or have used automated pipelines to assess pathogenicity of known variants. To determine the frequency of both medically actionable and nonactionable but medically relevant exome findings in the general population we assessed the exomes of 70 participants who have been extensively characterized over the past several years as part of a longitudinal integrated multiomics profiling study. We analyzed exomes by identifying rare likely pathogenic and pathogenic variants in genes associated with Mendelian disease in the Online Mendelian Inheritance in Man (OMIM) database. We then used American College of Medical Genetics (ACMG) guidelines for the classification of rare sequence variants. Additionally, we assessed pharmacogenetic variants. Twelve out of 70 (17%) participants had medically actionable findings in Mendelian disease genes. Five had phenotypes or family histories associated with their genetic variants. The frequency of actionable variants is higher than that reported in most previous studies and suggests added benefit from utilizing expanded gene lists and manual curation to assess actionable findings. A total of 63 participants (90%) had additional nonactionable findings, including 60 who were found to be carriers for recessive diseases and 21 who have increased Alzheimer's disease risk because of heterozygous or homozygous *APOE* e4 alleles (18 participants had both). Our results suggest that exome sequencing may have considerably more utility for health management in the general population than previously thought.

## INTRODUCTION

Genome and exome sequencing play increasingly important roles in providing molecular diagnoses for Mendelian disease (Manolio et al. 2013); however, our understanding of the extent to which genome and exome sequencing can benefit healthy individuals is limited. Although a few previous studies have attempted to elucidate the utility of genome or exome sequencing in healthy cohorts or individuals (Chen et al. 2012; Xue et al. 2012; Gonzalez-Garay et al. 2013; Dewey et al. 2014, 2015; Johnston et al. 2015; Vassy et al. 2017; Reuter et al. 2018), more have identified “incidental” or “secondary” findings in cohorts completely or partly composed of individuals with a known or suspected genetic disease and their family members (Johnston et al. 2012; Dorschner et al. 2013; Lawrence et al. 2014; Tabor et al. 2014; Yang et al. 2014; Amendola et al. 2015; Jang et al. 2015; Jurgens et al. 2015; Jamuar et al. 2016; Tang et al. 2018). These studies have reached a wide range of conclusions regarding the rate at which Mendelian disease-causing variants are identified, in large part because of significant differences in their approaches to variant filtering and curation, the use of gene lists to limit potential findings, and the lack of any standardized framework or guidelines for assessing and reporting exome or genome sequencing variants in generally healthy individuals.

In 2015 the American College of Medical Genetics and Genomics (ACMG) published guidelines to standardize the classification of genomic sequence variants (Richards et al. 2015). These guidelines reinforce the necessity of expert manual curation for accurate variant classification. However, manual curation is labor intensive and has been estimated to take nearly an hour per variant (Dewey et al. 2014). Although most clinical exome sequencing labs now utilize ACMG guidelines or similar criteria for variant classification, most previously published research studies assessing medically relevant genome and exome findings have classified variants using guidelines that predated the now widely utilized ACMG guidelines or relied on in silico predictors and/or matching variants against publicly available databases rather than employing manual curation (Dorschner et al. 2013; Gonzalez-Garay et al. 2013; Lawrence et al. 2014; Tabor et al. 2014; Gambin et al. 2015; Jurgens et al. 2015; Dewey et al. 2016). However, avoiding the step of expert variant curation significantly impairs the ability to accurately classify variants, as in silico predictors lack accuracy and current publicly available databases for human genomic variants contain variants that are incorrectly classified as disease-causing (Thusberg et al. 2011; Dewey et al. 2014; Vail et al. 2015; Masica and Karchin 2016). Moreover, most previous research studies also restricted their analyses by searching for variants in a limited list of genes. However, restricting the search for medically relevant variants to a targeted gene list—for example, the commonly used list of 59 genes compiled by the ACMG to guide the return of secondary findings—limits findings to only a fraction of potential genes associated with Mendelian disease (Green et al. 2013; Kalia et al. 2017). Thus, studies that perform an extensive analysis of Mendelian risk in generally healthy individuals using ACMG guidelines have not been performed, and as such the expected rate of actionable findings in a general population cohort is not known. This information is important for understanding the value of incorporating genome and exome sequencing into health care for healthy patients.

In this research study we endeavored to address this issue by performing an in-depth search for variants with potential medical significance in a group of 70 unrelated adult volunteers enrolled in a longitudinal wellness study. Our analysis included variants in all genes previously associated with Mendelian genetic diseases in the Online Mendelian Inheritance in Man (OMIM) (Hamosh et al. 2005) database or on the list of 59 ACMG genes. We demonstrate that the frequency of actionable variants is relatively high (17%). In addition we identified highly validated pharmacogenetics variants and *APOE* status. These results were reported back to participants by a genetic counselor in accordance with their expressed preferences for the types of results they would like to receive.

## RESULTS

### Participant Demographics

The exomes of 70 participants were analyzed. The participants were all generally healthy at the time of enrollment, with the exception of four diabetics, three of whom were previously diagnosed and are being treated, and one with diabetes detected at the time of enrollment because of an HbA1c ≥ 6.5%. Twenty out of 70 participants (29%) were prediabetic (defined by a HbA1c between 5.7% and 6.5%), which is similar to the general population prevalence of prediabetes (National Diabetes Statistics Report 2014). Participant characteristics are summarized in Table 1. They represented a range of self-reported ethnic backgrounds, including 48 Caucasian, eight Southeast Asian, six Indian, five African–American, and three Hispanic participants. Thirty-six participants were men and 34 were women. Their ages ranged from 34 to 76 yr old with a median age of 57. Fifty-five participants consented to make their sequences public—they are available at http://ihmpdcc.org/resources/osdf.php. Sixty-seven participants consented to include their sequencing data in dbGAP.

**Table 1.** Participant demographics

	Range (Median)
Age	34–76 (57)
Ethnicity	No. of Participants (% of cohort)
Caucasian	48 (69%)
Southeast Asian	8 (11%)
Indian	6 (9%)
African–American	5 (7%)
Hispanic	3 (4%)
Gender	No. of Participants (% of cohort)
Male	36 (51%)
Female	34 (49%)

### Exome Results

**Figure 1.**
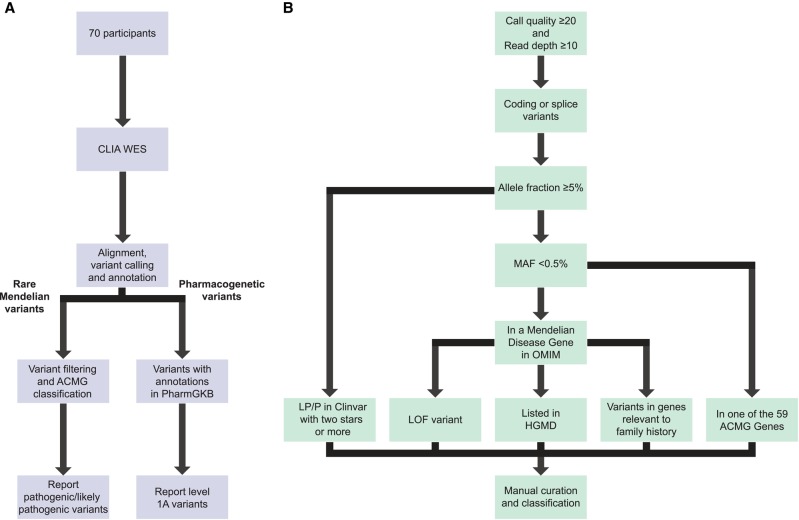
(*A*) Workflow; (*B*) variant filtering and curation. (*A*) A high-level overview of the workflow from exome sequencing through variant filtering and curation and reporting. (*B*) A more detailed description of the variant filtering criteria used to determine which variants would undergo manual curation.

The gene coding regions were sequenced using an enhanced exome sequencing strategy that provides comprehensive coverage of coding regions as well as additional genomic regions of interest (Patwardhan et al. 2015) (see Methods and Fig. 1A for workflow). A range of 149,311 to 262,804 variants was called per exome. Following the filtering steps described in Figure 1B, a total of 1464 variants were reviewed and further filtered manually as described in methods. A total of 680 variants (an average of 9.7 per participant) underwent manual curation using ACMG guidelines (Richards et al. 2015). Of these, 55 variants were classified as pathogenic and 95 as likely pathogenic. The remainder were classified as variants of unknown significance (VUSs), likely benign, or benign. The details of variant classification are presented in Table 2.

**Table 2.** Variant classifications

Variant call	Number of variants (average per participant)
Pathogenic	55 (0.8)
Likely pathogenic	95 (1.4)
Variant of unknown significance (VUS)	446 (6.4)
Likely benign	66 (0.9)
Benign	13 (0.2)
Reviewed and not classified^a^	793 (11.3)

^a^Variants were not classified if viewing the aligned reads suggested the variant was an artifact; if variants in that gene are expected to cause serious, highly penetrant disease at a young age and the participant did not have the associated phenotype (variants were only removed when the patient had a genotype that would be expected to cause disease were the variant pathogenic—i.e., homozygous for a recessive disease or heterozygous for a dominant disease); or if they were observed in >0.5% of a subpopulation in the ExAC or 1000 Genomes databases but passed the upstream MAF filter because the overall population MAF was <0.5%

**Figure 2.**
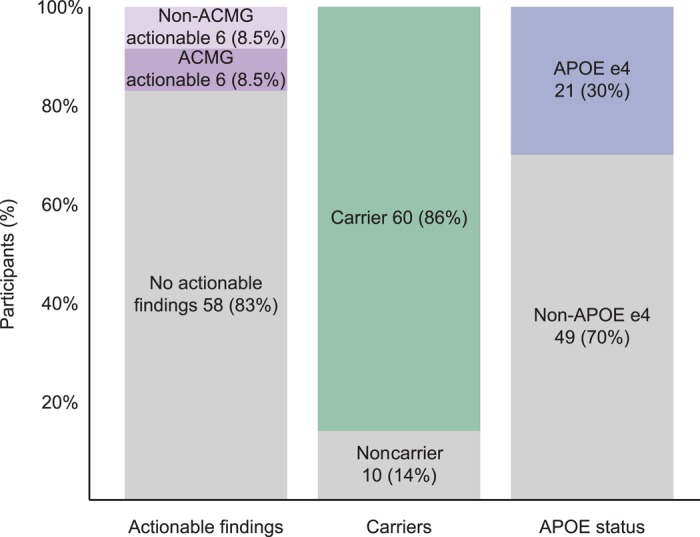
Actionable and nonactionable exome findings. The percentage of participants in whom each category of finding was identified. These include actionable findings, which are defined as likely pathogenic or pathogenic variants in genes associated with diseases that are moderately to highly penetrant, the identification of which was likely to result in altered medical management in the form of treatment, screening, or preventative measures, as described in published guidelines. The second category of findings represented is carrier status, which includes participants in whom at least one heterozygous likely pathogenic or pathogenic variant was identified in a gene associated with an autosomal recessive condition. The final category of finding is *APOE* status. The “APOE e4” designation includes all participants who had either one or two e4 alleles.

As expected, the vast majority of likely pathogenic and pathogenic variants identified in the cohort was located in genes associated with autosomal recessive diseases; therefore, the participants were considered heterozygous carriers who, in most cases, were unlikely to manifest symptoms. However, actionable pathogenic or likely pathogenic variants were identified in 12 participants (see Fig. 2). These variants were primarily in genes associated with autosomal dominant disease, although one pathogenic variant was in *MUTYH* (MIM: 604933)—a gene which is associated with autosomal recessive *MUTYH*-associated polyposis (MIM: 608456)—but for which heterozygotes are known to be at increased lifetime colon cancer risk (5.6% for female heterozygotes and 7.2% for male heterozygotes by age 70; higher for patients with a first-degree relative with colon cancer) (Win et al. 2014). Because of this increased risk, the National Comprehensive Cancer Network (NCCN) has issued screening guidelines for patients with heterozygous disease-causing variants (National Comprehensive Cancer Network 2016). Therefore, we considered this variant actionable. The actionable variants lie in 10 distinct genes (Table 3) and include five variants classified as pathogenic with strong evidence suggestive of a causative role in disease as per ACMG classification guidelines. Five were classified as likely pathogenic, and one variant that was identified in two individuals was classified as a risk allele. The risk allele—in the *APC* gene (MIM: 611731)—is a well-studied founder variant in the Ashkenazi Jewish population that the NCCN has described as a moderate risk allele for colon cancer and for which it has issued screening guidelines for individuals who are heterozygous for this variant (Boursi et al. 2013; Liang et al. 2013; National Comprehensive Cancer Network 2016). In total, 12 of the 70 individuals in the cohort (17% [95% CI 8%–26%]) had medically actionable likely pathogenic or pathogenic variants identified (see Table 3 for the complete list of actionable variants). Of the 12 variants, six reside in the 59 genes reported as actionable in the most recent ACMG guidelines regarding incidental findings (Green et al. 2013; Kalia et al. 2017). These include heterozygotes for likely pathogenic and pathogenic variants in the highly penetrant cancer risk genes *BRCA1* (MIM:113705), which is associated with hereditary breast and ovarian cancer (MIM: 604370), and *SDHB* (MIM: 185470), which is associated with hereditary paraganglioma and pheochromoctytoma (MIMs: 115310, 171300). The remaining six variants reside in genes that are not included in the ACMG guidelines but that are associated with medically actionable disease as defined in the methods.

**Table 3.** Medically actionable exome findings

	Gene	Chromosome	HGVS DNA	HGVS protein	Inheritance	Variant type	Variant call/associated disease	ACMG evidence (Richards et al. 2015)
Genes on the ACMG List^a^	*SDHB*	1	NM_003000; c.71dupA	p.A25fs	Dominant	Frameshift insertion	Likely pathogenic for hereditary paraganglioma and pheochromocytoma	PSV1 PM2^g^
	*SDHB*	1	NM_003000; c.137G>A	p.R46Q	Dominant	Missense	Likely pathogenic for hereditary paraganglioma and pheochromocytoma	PS3 (Kim et al. 2015; Saxena et al. 2016) PS4 (Gimenez-Roqueplo et al. 2002; Benn et al. 2003; Kimura et al. 2014)^d^ PP3 PP5
	*APC* (2)	5	NM_001127510; c.3920T>A	p.I1307K^c^	Dominant	Single nucleotide variant	Risk allele for colon cancer	PS3 (Laken et al. 1997) PS4 (Frayling et al. 1998; Woodage et al. 1998; Stern et al. 2001; Boursi et al. 2013)
	*BRCA1*	17	NM_007294; c.4689C>G	p.Y1563*	Dominant	Nonsense	Pathogenic for hereditary breast and ovarian cancer	PSV1 PS4 (Shih et al. 2002; Turkovic et al. 2010; Pern et al. 2012)^d^ PM2^g^
	*MUTYH*	1	NM_012222; c.724C>T	p.R242C	Recessive^b^	Missense	Pathogenic for familial adenomatous polyposis (increased colon cancer risk in heterozygotes)	PS3 (Ruggieri et al. 2012; Komine et al. 2015) PS4 (Miyaki et al. 2005; Olschwang et al. 2007)^d^ PM2^g^ PM3 (Ruggieri et al. 2012)
Genes not on the ACMG List^a^	*ABCC8*	11	NM_000352; c.1562G>A	p.R521Q	Dominant or Recessive	Missense	Likely pathogenic for AD hyperinsulinemia	PS4 (Calabria et al. 2012; Snider et al. 2013; Stranks et al. 2016)^d^ PM2^g^ PM5 (Banerjee et al. 2011) PP2^e^
	*HNF1A*	12	NM_000545; c.476G>A;	p.R159Q	Dominant	Missense	Pathogenic for maturity-onset diabetes of the young (MODY)	PS3 (Tonooka et al. 2002) PS4 (Vaxillaire et al. 1997; Yamada et al. 1999; Bazalová et al. 2010) PM2^g^ PP1 (Vaxillaire et al. 1997; Costa et al. 2000) PP3
	*PROS1*	3	NM_000313; c.586A>G	p.K196E	Dominant	Missense	Pathogenic for protein S deficiency	PS3 (Hayashi et al. 1994; Banno et al. 2015) PS4 (Hayashi et al. 1994; Kimura et al. 2006; Miyata et al. 2009; Ikejiri et al. 2010; Hayakawa et al. 2011; Neki et al. 2011; Huang et al. 2016) PP5
	*CHEK2*	22	NM_001005735; c.1497dupA	p.E500fs	Dominant	Frameshift insertion	Likely pathogenic for *CHEK2*-related cancers, including breast cancer	PSV1 PM2^g^
	*RBM20*	10	NM_001134363; c.1898C>T	p.P633L	Dominant	Missense	Likely pathogenic for dilated cardiomyopathy	PM1 (Li et al. 2010) PM2^g^ PP1^f^ PP3
	*SLC7A9*	19	NM_014270; c.544G>A	p.A182T	Dominant or Recessive	Missense	Pathogenic for AD cystinuria	PS3 (Font et al. 2001) PS4 (Font et al. 2001; Halbritter et al. 2014)

A (2) indicates that two participants were heterozygous for this variant.

All variants in Table 3 were found in heterozygous state.

^a^The American College of Medical Genetics and Genomics (ACMG) created a gene list to guide the return of incidental findings for patients undergoing exome or genome sequencing (Green et al. 2013; Kalia et al. 2017).

^b^The *MUTYH* gene is included on the ACMG gene list; however, as per ACMG guidelines only compound heterozygous or homozygous variants in this gene should be reported as incidental findings (Green et al. 2013). Heterozygotes for *MUTYH* variants are, however, at increased risk for developing colon cancer, and the National Comprehensive Cancer Network (NCCN) recommends increased screening for heterozygotes (Win et al. 2014; National Comprehensive Cancer Network 2016).

^c^This *APC* variant does not cause traditional familial adenomatous polyposis, but rather has been shown to increase risk for colon cancer, and the NCCN recommends increased surveillance for individuals who are heterozygous for this specific variant (Boursi et al. 2013; Liang et al. 2013; National Comprehensive Cancer Network 2016).

^d^This evidence was used as “moderate” level evidence as per ACMG guidelines.

^e^The *ABCC8* gene was determined to have low tolerance for benign missense variation using a http://genic-intolerance.org (Petrovski et al. 2013).

^f^Unpublished data.

^g^The determination of whether to apply PM2 criteria was made on a case-by-case basis for variants that were present in population databases but at low frequencies. Considerations included the MAF of the variant, the reported prevalence of the associated condition, and the reported penetrance of the condition.

### Patients with Personal or Family Medical History Consistent with their Variant

At least five individuals have personal or family medical histories consistent with the presence of their variants. A 46-yr-old female with elevated glucose and a significant family history of diabetes was found to be heterozygous for a pathogenic *HNF1A* (MIM: 142410) variant. *HNF1A* variants cause autosomal dominant maturity-onset diabetes of the young (MODY; MIM: 600496), a form of monogenic diabetes that is often misdiagnosed as type 1 or type 2 diabetes, as was the case in this participant, who was incorrectly diagnosed with type 2 diabetes in her early 30s.

Patients with diabetes caused by *HNF1A* variants most often develop diabetes in late adolescence or early adulthood, but a significant subset of cases start after age 25 (40% of cases in one large study; Bellanné-Chantelot et al. 2008). In addition to her own history of diabetes, the participant has a family history of diabetes spanning three generations, including her 42-yr-old sister, her mother who also developed diabetes in her early 30s, several maternal uncles, and her maternal grandfather. The participant underwent an oral glucose tolerance test (OGTT) at the beginning of the study, and her fasting plasma glucose rose from a starting value of 149 mg/dL to 347 mg/dL at 2 h—this large increase is also suggestive of diabetes caused by a pathogenic *HNF1A* variant, as is the participant's consistently low C-reactive protein levels (Gardner and Tai 2012). Compared to type 2 diabetes, diabetes caused by *HNF1A* variants is considerably more responsive to sulphonylurea drugs. Early recognition of *HNF1A*-MODY and subsequent initiation of sulphonylurea therapy may reduce the incidence of diabetic complications (Shepherd et al. 2009; Bacon et al. 2015). The participant reported that she had previously tried a sulphonylurea medication to manage her diabetes but was taken off of the medication because of significant hypoglycemia, which we hypothesize may be due to the sensitivity of *HNF1A* diabetics to sulphonylureas. It has been previously reported that patients with diabetes caused by *HNF1A* variants are fivefold more sensitive to sulphonylureas and typically need a much lower dose of the drug to prevent hypoglycemia (Pearson et al. 2003; Shepherd et al. 2009). The participant, who is currently managing her diabetes with a combination of three nonsulphonylurea oral medications, was referred to endocrinology to discuss potential changes to her treatment plan, and her three children also underwent genetic testing for the variant to inform diabetes screening regimens.

Another participant with a family history of dilated cardiomyopathy (DCM; MIM: 613172) was identified to be heterozygous for a likely pathogenic *RMB20* (MIM: 613171) variant. The variant has not been previously reported as associated with DCM but was prioritized for curation as a result of the participant's family history. The variant is in a hotspot for DCM-associated variants located in the RS domain of *RBM20* and is located in a codon adjacent to a series of five codons previously reported in DCM cases (Li et al. 2010). Because of family history, as well as low-normal ejection fraction on a follow-up echocardiogram, the participant began taking blood pressure–lowering medications as a preventative measure. The participant was referred to cardiovascular genetics clinic for further follow-up.

A 44-yr-old male participant with a significant family history of both type 1 and type 2 diabetes and a personal history of insulin-dependent diabetes diagnosed at age 34 was found to have an *ABCC8* variant previously reported to cause autosomal dominant hyperinsulinemia (ABCC8; MIM: 600509). The participant had no known history of hyperinsulinemia; however, reduced penetrance and variable expressivity are well-reported features of the dominant form of hyperinsulinemia (as opposed to recessive hyperinsulinemia, which is almost completely penetrant and tends to be severe) (Huopio et al. 2000; Thornton et al. 2003; Pinney et al. 2008). Interestingly, several previous studies have suggested a link between dominant hyperinsulinemia and young onset of diabetes, which has been hypothesized to be a result of progressive β-cell failure due to “burnout” or increased β-cell apoptosis due to elevated intracellular calcium concentration (Huopio et al. 2000, 2003; Thornton et al. 2003; Abdulhadi-Atwan et al. 2008; Vieira et al. 2010; Kapoor et al. 2011; Baier et al. 2015), although this link has been refuted by at least one study (Pinney et al. 2008).

A 41-yr-old female participant was found to have a likely pathogenic frameshift variant in *SDHB*, a gene in which variants cause autosomal dominant hereditary paraganglioma and pheochromocytoma. The participant followed up in a cancer genetics clinic to have the variant confirmed and then began undergoing regular screening, including MRI, which identified early-stage papillary thyroid cancer. As a result, she underwent a hemithyroidectomy. Her sister was also tested and found to have the same variant. She has no other family history of *SDHB*-related cancers that we are aware of.

A pathogenic *PROS1* variant was identified in a female participant who had no personal history of the clotting events. *PROS1* variants cause increased risk for thrombophilia due to protein S deficiency. Preventative treatment is indicated in some patients, particularly those who already have a family history of thrombotic events (De Stefano and Rossi 2013). Oral contraceptives are also contraindicated in women with heterozygous *PROS1* variants, even in the absence of family history of thrombotic events (van Vlijmen et al. 2016). The participant did have a family history of transient ischemic attacks and strokes in her father and four of her father's five siblings. However, the genotypes of these family members are unknown, and other factors may be involved in the family history. For example, the family members with the history of clotting events also all had a history of elevated blood pressure, which can also lead to higher risk for clotting events.

### Non–Medically Actionable Findings

A total of 63 participants (90% of the cohort) were identified to have nonactionable findings (including carriers for recessive conditions and/or *APOE* e4 allele carriers—see Fig. 2). In 60 participants we identified 150 likely pathogenic and pathogenic variants in genes that cause autosomal recessive diseases (see Supplemental File 1 for a complete list of likely pathogenic and pathogenic variants). Most of these variants convey no health risks to carriers beyond reproductive risks, but there are exceptions. In addition to the *MUTYH* variant discussed earlier, pathogenic heterozygous *GBA* (MIM: 606463) variants were identified in two participants. *GBA* variants cause autosomal recessive Gaucher disease (MIMs: 608013, 230800, 230900, 231000, 231005), but like individuals affected with Gaucher disease, heterozygotes are also at significantly increased risk for Parkinson's disease (MIM: 168600) (Tayebi et al. 2003; Halperin et al. 2006; Alcalay et al. 2014). In addition, 21 participants were identified to be heterozygous or homozygous for the *APOE* (MIM: 107741) e4 allele, which is associated with increased lifetime risk for developing Alzheimer's disease (MIM: 104310) (Corder et al. 1993; Bertram et al. 2010). The *APOE* genotype was only disclosed in two cases in which participants specifically inquired about their status and had opted to receive both actionable and nonactionable findings on their consent form.

### Pharmacogenetic Variants

In addition to disease-causing variants we also assessed participant exomes for variants impacting response to drugs. Level 1A variants in PharmGKB (Whirl-Carrillo et al. 2012) have high confidence for affecting drug dose and/or side effects. The 70 exomes were examined for 28 rsIDs with level 1A classifications (extracted from pharmgkb.org in May 2017) (see Supplemental File 2 for a list of rsIDs). A range of one to six level 1A variants were identified per participant, with a median of three variants. Well-known examples include several variants in *CYP2C19* that are associated with altered metabolism or risk of side effects for drugs such as clopidogrel and amitriptyline, including rs9923231 and rs4244285 (Whirl-Carrillo et al. 2012). Thus, overall, the majority of our participants received potentially useful pharmacogenetic information.

## DISCUSSION

Although genome and exome sequencing have great potential for prediction of disease risk for healthy individuals, to date most studies attempting to establish the rate of actionable findings in such populations have focused only on limited gene lists such as the ACMG gene list (Green et al. 2013; Kalia et al. 2017), used automatic pipelines with limited or no manual variant curation, or performed variant curation without standardized guidelines. Thus, the frequency with which healthy individuals might learn actionable information from their genome sequence using current guidelines and based on comprehensive assessment is not known.

Our study attempts to contribute to a baseline understanding of the potential for actionable exome findings in a general population cohort. We found that a larger percentage of participants had actionable findings (17%, or 11% if the moderately penetrant variants in *MUTYH*, *CHEK2*, and *APC* are excluded) than reported in most previous studies, which typically range from ∼0.5% to 5%. A 2017 study by Vassy et al. (2017) reported disease-causing variants in a much more extensive list of genes and described a similar rate of actionable findings to ours (22%) (albeit utilizing different methodology), as did a 2018 study by Reuter et al. which found a rate of 25% (Reuter et al. 2018). Both their results and ours suggest that although variants in the 59 ACMG genes are actionable and therefore clinically relevant, there is much to be gained from examining a more comprehensive list of genes, as the list of 59 genes represents only a fraction of those known to cause Mendelian disease in humans.

In addition to likely pathogenic and pathogenic variants in genes on the ACMG list, we identified pathogenic or likely pathogenic variants in six other genes not on the ACMG list that have medical relevance; of these, four (*HNF1A*, *RBM20*, *ABCC8*, and *PROS1*) were found in participants who had personal and/or family medical history consistent with pathogenicity. One participant with an actionable variant in a gene on the ACMG list, *SDHB*, also had a phenotype consistent with her variant.

Identification of two of these actionable variants (*RBM20* and *SDHB*) led to screening that identified associated findings (evidence of DCM and papillary thyroid cancer, respectively) and may have a significant impact on these participants’ long-term health. Some participants with variants identified in actionable genes did not have a personal or family history of the associated phenotype, which may be because of reduced penetrance. Alternatively, the pathophysiology may not yet have arisen.

We also identified pathogenic and likely pathogenic variants in other noteworthy genes. As previously noted, one participant was found to carry a likely pathogenic *MUTYH* variant. Current ACMG guidelines for the reporting of incidental findings recommend only reporting compound heterozygous or homozygous variants in *MUTYH*, as *MUTYH*-associated polyposis is considered a recessive disease (Green et al. 2013; Kalia et al. 2017). However, individuals with heterozygous *MUTYH* variants are at an increased risk of colon cancer, and the NCCN has recommended that they undergo more frequent colonoscopies starting at an earlier age than the general population recommendations (National Comprehensive Cancer Network 2016). In one male participant we identified a pathogenic *CHEK2* variant, which leads to a dominantly inherited moderate lifetime risk for cancers including breast and colorectal (Meijers-Heijboer et al. 2002; Xiang et al. 2011). *CHEK2* is not on the ACMG list, but the NCCN recommends increased cancer screening starting at younger ages for patients with *CHEK2* variants (National Comprehensive Cancer Network 2017). Identification of such variants can also alert family members to their potential cancer risk, which for female relatives of our participant found to carry his same variant would include a significant (potentially more than twofold) increased breast cancer risk (CHEK2 Breast Cancer Case-Control Consortium 2004). Both of these participants were referred to a cancer genetics clinic for follow-up.

Although actionable findings were the focus of our study, participants also had the option of receiving nonactionable results, and a number of studies have suggested that many patients and research participants do want to learn about incidental or secondary findings that are not medically actionable—for example, the genetic risk for developing adult-onset neurodegenerative conditions such as Alzheimer's disease or Parkinson's disease—and that anxiety or depression is not increased as a result of learning about these risks (Green et al. 2009; Bemelmans et al. 2016). Qualitative research on this subject has suggested many individuals find this information actionable in other (nonmedical) ways and express that they would live their lives differently if they knew they were at increased risk of developing such a condition or would prepare for developing the disease (Clift et al. 2016; Yushak et al. 2016). Of note, the ACMG has recommended that clinicians not order *APOE* testing for the purpose of predictive testing for Alzheimer's disease, as heterozygous or homozygous e4 alleles are neither necessary nor sufficient to cause Alzheimer's disease (https://www.acmg.net/docs/ACMG_ChoosingWisely_Final.pdf). We felt that the potential value of having this information to participants outweighed the risks, and participants who expressed an interest in receiving their *APOE* results were counseled extensively that *APOE* status represents a risk factor for Alzheimer's disease and is not definitive. Our study identified 21 participants with one or two copies of the *APOE* e4 allele, which significantly increases lifetime risk for developing Alzheimer's disease (Corder et al. 1993; Bertram et al. 2010). This information was reported back only to the two participants who specifically requested their *APOE* status. Similarly, we identified two heterozygous carriers of pathogenic *GBA* variants, and although *GBA* heterozygotes will not develop Gaucher disease—an autosomal recessive lysosomal storage disease—they are at increased risk for developing Parkinson's disease (Tayebi et al. 2003; Halperin et al. 2006; Alcalay et al. 2014). We reported this information back to participants who opted to learn all medically relevant findings. For the *GBA* heterozygotes in our study as well as the 60 carriers of disease-causing variants in other genes implicated in recessive disease, this information can also alert families to potential reproductive risk and lead to carrier testing for their partners or adult children.

In addition to using an expanded gene list, another potential reason for the difference in our rate of actionable findings in comparison to previous studies is the use of manual variant curation. Although it is now standard in clinical exome laboratories to classify variants using manual curation based on ACMG guidelines, this is not the methodology used in much of the existing *research* that identified actionable findings in exomes and reported on the rates of actionable findings. (This is in large part because the ACMG variant classification guidelines have only existed since 2015, so although commercial laboratories were employing manual classification prior to 2015 it was not with these standardized guidelines.) In addition to limiting secondary findings to variants within a narrow gene list, researchers attempting to identify genome and exome secondary findings have in many cases mitigated the curation workload by forgoing variant curation altogether. A number of previous research studies assessing secondary findings have either completely or primarily relied on a combination of in silico predictors and/or variant databases such as ClinVar (Landrum et al. 2014) and HGMD (Stenson et al. 2003) to identify variants of interest rather than employing manual curation and classification guidelines (Gonzalez-Garay et al. 2013; Tabor et al. 2014; Gambin et al. 2015; Dewey et al. 2016). There are, of course, exceptions—a few recent studies have assessed rates of actionable findings utilizing the ACMG variant classification criteria (Vassy et al. 2017; Jain et al. 2018; Reuter et al. 2018), although the expansive difference between the rates of actionable findings in these papers (0.59%–25%) is illustrative of the lack of a standardized approach.

Testing well-known, previously classified missense variants with the commonly used in silico predictors SIFT and PolyPhen yields accuracy ranging from 62% to 78% (Masica and Karchin 2016). Splice site predictors are only slightly more accurate (Vreeswijk et al. 2008; Houdayer et al. 2012). Although improving, the majority of variants in ClinVar have not undergone expert review, and classifications are sometimes based on incomplete or outdated evidence and/or were classified without applying stringent criteria. Increasingly, the variants listed in ClinVar have been submitted by CLIA-certified laboratories utilizing the ACMG classification guidelines; however, there are still many entries with conflicting interpretations of pathogenicity that were submitted by OMIM (and are therefore based on literature, sometimes from a single paper claiming to have identified a disease-causing variant) that were classified before larger population databases such as ExAC became available in 2015, or that were submitted by laboratories that did not utilize established guidelines for variant classification.

Similarly, variants listed as disease mutations (DMs) in the HGMD frequently do not meet criteria to be classified as likely pathogenic or pathogenic. Dewey et al. (2014) found that only one-fourth of the HGMD DM variants they identified in their cohort were classified by experienced curators as likely pathogenic or pathogenic. This evidence supports the need for manual variant curation to accurately classify variants. As most previous research studies reporting on the rate of incidental or secondary actionable findings in exome sequencing either performed limited variant curation and/or limited their gene list significantly, this is among the first studies to fully explore the rate of actionable findings that might be expected in a general population cohort.

The degree of participant family and personal medical history available to us in this study may also have contributed to the differences between our rate of actionable findings compared to other studies. In two cases (*HNF1A* and *RBM20*) we were able to broaden our search for actionable findings based on a specific phenotype reported in the participant's family, which may have increased the rate of findings. However, we also were sometimes able to rule out pathogenicity of a variant in a highly penetrant gene based on the absence of a phenotype in our participant, which may have lowered the rate.

Our study had several limitations. Among them, we used a minor allele frequency cutoff of 0.5% when filtering variants for further curation. Although we attempted to “rescue” common founder variants that are more common than 0.5% by capturing variants in ClinVar as pathogenic/likely pathogenic with two or more stars, we still certainly missed disease-causing variants that are more common than 0.5% but were either not in ClinVar or did not meet the two-star threshold. We may have also discarded disease-causing variants because they were in highly penetrant genes and the participant did not have the associated phenotype. Although we attempted to be conservative when discarding variants for that reason, we cannot rule out the possibility that participants had disease-causing variants in the expected inheritance pattern to cause disease but for unknown reasons did not manifest with symptoms—a phenomenon described recently by Chen et al. (2016). Other filtering cutoffs also likely limited the number of disease-causing variants identified, as did the use of the OMIM gene list. The OMIM list may not include the most recently discovered Mendelian disease associations and provides those genes with highly penetrant variants typically identified through familial studies; because disease-causing variants have a wide range of penetrance, this definition can be subjective. Our understanding of penetrance in many disease genes is based largely on studies of families known to be affected with disease, so in the future we may learn that penetrance is lower for individuals without family histories of disease who have actionable pathogenic variants described in this study. It is important to note that as more disease-causing variants are identified and their penetrance known, more precise genome interpretation will ensue, and it also is plausible that the number of actionable variants discovered in an individual's genome will grow.

Another limitation is the potential for false-positive interpretations of variants. Even when variants are accurately classified as pathogenic or likely pathogenic based on available data, it is not a guarantee that they are truly disease-causing. Reporting likely pathogenic variants in particular reduces specificity and negatively impacts positive predictive value. This challenge is exacerbated when a test such as exome sequencing is used for population screening because the low prevalence of many of the conditions screened for means that even small reductions in specificity greatly increase false positives (Adams et al. 2016). False-positive results have the potential to cause significant harm, including psychological distress, resources wasted on unnecessary testing and/or interventions, and the potential for physical harm from unneeded treatments or procedures.

The ACMG guidelines for variant classification were designed first and foremost to be applied in the context of cases of suspected genetic diseases, but we applied these guidelines outside of their intended use (in generally healthy individuals, sometimes as a means of classifying moderate-penetrance variants that might best be considered risk alleles) because these guidelines are the most rigorous, structured option we are aware of for variant classification. We believe this application of the ACMG classification guidelines is similar to that of many laboratories that use the guidelines to classify secondary findings; however, as the ACMG described in Richards et al. (2015), extra caution must be applied when using these guidelines in the context of healthy individuals, as variants are less likely to be disease-causing than when identified as part of targeted disease testing and because penetrance of identified pathogenic variants may be further reduced in such contexts.

It is important to note that the application of the term “actionable” is subjective. In this study we chose to use the term broadly—for example, describing variants as actionable even though the primary relevance may be for immediate family members, not for the proband. For example, our *BRCA1*-positive patient has been advised to undergo the relevant cancer screening for males, but the more significant risks with this variant are for his close female relatives. Our patient with the *ABCC8* variant already has diabetes, so other significant risks to consider would be for hyperinsulinemia and possibly diabetes risk in his children.

Also, we note that our cohort size (70) is small and larger studies will be needed to determine if the rate of actionable findings identified in our cohort also applies to larger populations.

One frequently raised concern surrounding the idea of expanded incidental/secondary finding gene lists is the potential for increased anxiety or stress and unnecessary and costly follow-up care for patients/participants receiving results that may not change their health-care management and/or may be associated with small increases in disease risk or have uncertain significance. This is particularly expected to be an issue when including VUSs, nonactionable results such as increased risk for diseases including Alzheimer's disease, or variants in genes with reduced penetrance. Expanding gene lists for the return of secondary findings would increase the chance for these types of findings to be identified and raise complicated practical and ethical questions regarding how best to balance participant autonomy and minimize negative outcomes. In this study, we chose not to return VUSs to participants because of their limited utility in a generally healthy population. We did return moderate penetrance variants such as the *APC* I1307K variant and the heterozygous *MUTYH* variant described above because of their actionability, and there are NCCN guidelines for the management of patients with these variants (National Comprehensive Cancer Network 2016). However, it is important to note that the decision to return information about variants with low penetrance is subjective and depends on several factors, including the ease, cost, and frequency of the screening and the severity of the potential disease.

We also returned nonactionable findings when participants specified that they wanted to learn such information, as the limited literature available suggests that returning non–medically actionable findings is associated with limited negative outcomes and has potential utility to participants (Lewis et al. 2016; Sanderson et al. 2017). However, significantly more research is needed to guide the approach to these types of findings, including research into the utility of such findings for patients and their care providers, as well as research into the long-term impacts of returning these types of results.

## CONCLUSIONS

We demonstrate that exome sequencing of participants in a longitudinal wellness study reveals medically relevant information in a considerable fraction of the population. More research is needed to better understand (1) the breadth of medically actionable variants, particularly as more data become available, (2) which other types of results patients and health-care providers find useful, (3) the costs and benefits of returning more extensive secondary findings to patients undergoing exome or genome sequencing, and (4) how best to tailor reporting criteria to maximize utility and minimize harm from false-positive and false-negative results. Nonetheless, we conclude that the rate of actionable findings found in our cohort (17%) suggests that employing manual variant curation and expanded gene lists may enhance the identification of actionable variants in exomes—genomic information that may ultimately lead to improved disease risk prediction and prevention, improved screening, and/or early disease detection.

## Methods

### Recruitment and Study Population

Participants were enrolled as part of Stanford's iPOP (Integrated Personal Omics Profiling) research study (IRB 23602), which entails longitudinal multiomics profiling of a cohort of unrelated adult volunteers enriched for prediabetics. The iPOP study has been described previously (Chen et al. 2012; The Integrative Human Microbiome Project 2014). All research participants received genetic counseling by a medical geneticist or genetic counselor prior to enrollment and signed a consent form approved by the Stanford University Institutional Review Board. Participants were able to opt in or out of receiving exome results and, if they opted in, were also given the option of selecting whether they wanted only actionable results or all results with medical relevance.

### Exome Sequencing

Exome sequencing was performed on 70 individuals. Briefly, DNA was isolated from blood using Gentra Puregene Kits (QIAGEN) according to the manufacturer's protocol. Exome sequencing was performed at Personalis—a CLIA- and CAP-accredited facility—using the ACE Clinical Exome Test, which covers exomes in a more comprehensive fashion (Patwardhan et al. 2015) and additional genomic regions of interest. Paired-end sequencing with 100-bp reads and average coverage of 70× was used. Variants were called using the HugeSeq pipeline (Lam et al. 2012), which used GATK 3.1.7-7 (McKenna et al. 2010). CNVs and mobile elements were not called as part of this analysis.

### Variant Filtering and Analysis

The overall workflow is depicted in Figure 1A. Two types of genomic results were assessed—rare variants in known Mendelian disease genes and variants with pharmacogenetic annotations in the PharmGKB database (Whirl-Carrillo et al. 2012). Rare variants were filtered according to the steps depicted in Figure 1B. Initially variants were filtered based on confidence metrics including Phred scores (minimum 20) and read depth (minimum 10). To be included in the analysis variants had to be coding or canonical splice variants. Variants were also excluded if they had a minor allele frequency of >0.5% in the 1000 Genomes database (The 1000 Genomes Project Consortium et al. 2015) or Exome Aggregation Consortium (ExAC) database (Lek et al. 2016). We then removed variants that did not appear in one of the 3659 genes in the OMIM database categorized as a gene associated with Mendelian disease (downloaded January 2016—Supplemental File 4) or on the list of 59 genes in which the ACMG recommends reporting incidental findings (Green et al. 2013; Kalia et al. 2017). OMIM genes for which the only disease annotations are those OMIM categorized as exceptions to clear-cut Mendelian disease-causing genes—including nondisease genes (usually genetic variants leading to abnormal laboratory test values not associated with actual disease phenotype) indicated in the OMIM gene entry by brackets, genes associated with multifactorial disease indicated in the OMIM gene entry by braces, and genes for which the disease association is provisional indicated in the OMIM gene entry by a question mark—were not included in our analysis unless they were on the ACMG list. Additional filtering was performed in several ways.

1. Variants for which manual examination of the aligned reads indicated a likely sequencing error were removed.
2. Variants expected to cause serious, highly penetrant disease at a young age and for which we had sufficient medical history to be confident that the participant did not have the associated phenotype were removed. If a gene was reported to exhibit age-related penetrance and our participant was younger than the age at which complete penetrance was reported, we did not exclude the variant. Similarly, we kept variants in genes where the phenotype may be unknown to the patient or not present in the medical records because it was mild, required imaging to detect, etc. Variants were only removed when the patient had a genotype that would be expected to cause disease were the variant pathogenic (i.e., homozygous for a recessive disease or heterozygous for a dominant disease); our experience revealed that these were usually artifacts (see Supplemental File 3 for a list of variants removed for this reason).
3. When the curators determined there was insufficient evidence that the gene in which the variant resided was associated with disease (e.g., it was an association based solely on genome-wide association studies or only one paper with few affected individuals), the gene was removed.
4. When the minor allele frequency of the variant in the 1000 Genomes or ExAC database was >0.5% in a subpopulation but had initially passed filtering because the overall population minor allele frequency was below that cutoff, the variant was removed. Variants with subpopulation MAFs of >0.5% were, however, included in the analysis if the highest subpopulation MAF was calculated based on a relatively small population or there were a very small number of carriers (e.g., if the MAF was based on one out of 500 alleles, we would use the next highest subpopulation MAF instead).

The following categories of rare variants then underwent manual curation and classification by a trained genetic counselor according to ACMG criteria for the classification of sequence variants: (1) variants of a type likely to cause loss of gene function (insertions and deletions, nonsense, splice), (2) variants with an exact match in the Human Genome Mutation Database (HGMD), and (3) coding or canonical splice-site variants in one of the 59 genes in which ACMG recommends reporting incidental findings (Richards et al. 2015; Kalia et al. 2017). To avoid missing common founder variants that are >0.5% MAF, we also “rescued” coding and splice variants that were above the MAF threshold but were classified in ClinVar as pathogenic or likely pathogenic with two or more stars (indicating multiple submitters). These variants then underwent manual curation as described above.

Participants had varying degrees of personal and family medical history available for the curators to take into consideration when classifying variants. For some participants this information was limited to a medical history intake form and/or basic medical records; for others much more extensive medical history and/or a three-generation pedigree were available. Additional variants were sometimes curated when they were identified in genes associated with a potentially Mendelian disease in the participant's family or personal medical history.

Participants in whom medically significant likely pathogenic or pathogenic variants were identified were encouraged to discuss the results with their physician and, when necessary, referred to a genetics clinic for follow-up and testing to confirm the variant. Participants were given the option at the time of consent of selecting whether they would like to receive genomic results and, if so, whether they would prefer actionable results only or all medically relevant results identified. Actionable results were defined as likely pathogenic or pathogenic variants in genes associated with diseases that are moderately to highly penetrant, the identification of which was likely to result in altered medical management in the form of treatment, screening, or preventative measures, as described in published guidelines. Additionally, nonactionable findings with medical relevance were returned to participants who opted to receive them during the consent process. These results included likely pathogenic and pathogenic heterozygous variants in genes implicated in recessive diseases, as well as likely pathogenic and pathogenic variants in genes associated with diseases such as Parkinson's disease or Alzheimer's disease, for which limited or no highly effective treatment or preventative measures are available. Variants of unknown significance were not returned to participants, as they have the potential to cause participants anxiety and are usually not actionable. Pathogenic and likely pathogenic variants were reviewed by two genetic counselors and a medical geneticist. Variants were not confirmed using an alternative method such as Sanger sequencing before being returned to participants, and therefore participants were counseled with all returned results that there was a possibility that the variants were called in error. Results were then reported back to participants by a genetic counselor in accordance with their stated preferences.

Participants’ genotypes were also examined for common SNPs with pharmacogenetic annotations that reached a level 1A classification in the PharmGKB database (Whirl-Carrillo et al. 2012). Level 1A variants represent those with the highest level of validation.

## Additional Information

### Data Deposition and Access

Data from participants who consented to make their sequences completely public are available at http://ihmpdcc.org/resources/osdf.php. Variants that appear in this manuscript have been deposited in ClinVar (http://www.ncbi.nlm.nih.gov/clinvar/) and can be found under accession numbers SCV000853086–SCV000853096.

### Ethics Statement

The study was approved by the Stanford University Institutional Review Board (approval number 23602). All patients provided written informed consent and the study was conducted in accordance with the Declaration of Helsinki.

### Acknowledgments

The authors would like to thank the Stanford Genetics Bioinformatics Service Center, Sophia Schüssler-Fiorenza Rose, and Jenny Yong for computational, informatics, and graphics support, as well as the volunteers who participated in our study.

### Author Contributions

This study was conceived and designed by S.R., O.D.-R. and M.P.S. Bioinformatics support was provided by M.R.S., D.S., H.L.R., and J.D. Data analysis was performed by S.R. and O.D.-R. with guidance from J.A.B. Assistance with participant recruitment and data collection was provided by W.Z., P.L., E.C., C.C., and T.M. The manuscript was written by S.R. and O.D.-R. with support from W.Z., H.L.R., J.D., T.M., L.M.S., J.A.B., and M.P.S. All authors read and approved the manuscript.

### Funding

Our work was supported by grants from the National Institutes of Health (NIH) Common Fund Human Microbiome Project (HMP) (1U54DE02378901) (M.P.S. and T.M.) and the American Diabetes Association (grants 1-14-TS-28 and 1-11-CT-35) (T.M.). M.R.S. and H.L.R. are supported by grants from the Swiss National Science Foundation (SNSF: P300PA_161005, P2GEP3_151825, M.R.S.; P300PA_164703, H.L.R.). J.D. is funded by the Mobilize Center (grant NIH U54 EB020405). This work was also supported by a gift from the Forbes Family Fund and by Chamath Palihapitiya and Brigette Lau.

### Competing Interest Statement

M.P.S. is a founder and member of the science advisory board of Personalis, SensOmics, and Qbio and a science advisory board member of Genapsys. L.M.S. is a founder and member of the science advisory board of Sophia Genetics and Levitas. S.R. is a consultant for Qbio.

## Supplementary Material

Supplemental Material
