# Supplementary material for: High-frequency actionable pathogenic exome variants in an average-risk cohort
Source: Cold Spring Harb Mol Case Stud. 2018 Dec;4(6):a003178. doi: 10.1101/mcs.a003178 (PMC6318774; doi:10.1101/mcs.a003178)
Supplement: Supplemental Material [file supp_4_6_a003178__index.html]

High-frequency actionable pathogenic exome variants in an average-risk cohort — Supplemental Material 

# High-frequency actionable pathogenic exome variants in an average-risk cohort

## Supplemental Material

- Supplemental\_File\_1.xlsx
- Supplemental\_File\_2.xlsx
- Supplemental\_File\_3.xlsx
- Supplemental\_File\_4.xlsx
- Supplemental\_Legends.docx
