# Supplementary material for: High-frequency actionable pathogenic exome variants in an average-risk cohort
Source: Cold Spring Harb Mol Case Stud. 2018 Dec;4(6):a003178. doi: 10.1101/mcs.a003178 (PMC6318774; doi:10.1101/mcs.a003178)
Supplement: Supplemental Material [file supp_mcs.a003178_Supplemental_Legends.docx]

*Supplemental Files*

Supplemental File 1: Pathogenic and likely pathogenic variants identified in this study

Supplemental File 2: rsIDs for level 1A pharmGKB variants assessed in this study

Supplemental File 3: Variants in highly penetrant genes identified in unaffected participants (see Methods for details)

Supplemental File 4: OMIM gene list used in variant filter
